# *Giardia duodenalis* in common bile duct brushings

**DOI:** 10.1002/hsr2.177

**Published:** 2020-08-10

**Authors:** Abha Thakur, Haimanti Sarin

**Affiliations:** ^1^ Department of Pathology Medanta‐The Medicity Gurgaon Haryana India

We report a case of a 47‐year‐old male who presented for endoscopic biliary stent removal due to common bile duct (CBD) stricture (IgG4 related cholangitis). CBD brushing incidentally revealed trophozoites of *Giardia duodenalis* (*G. duodenalis*) with characteristic pear/tear drop shape, bilaterally symmetric nuclei and flagella (Figure 1).

**Figure 1:**
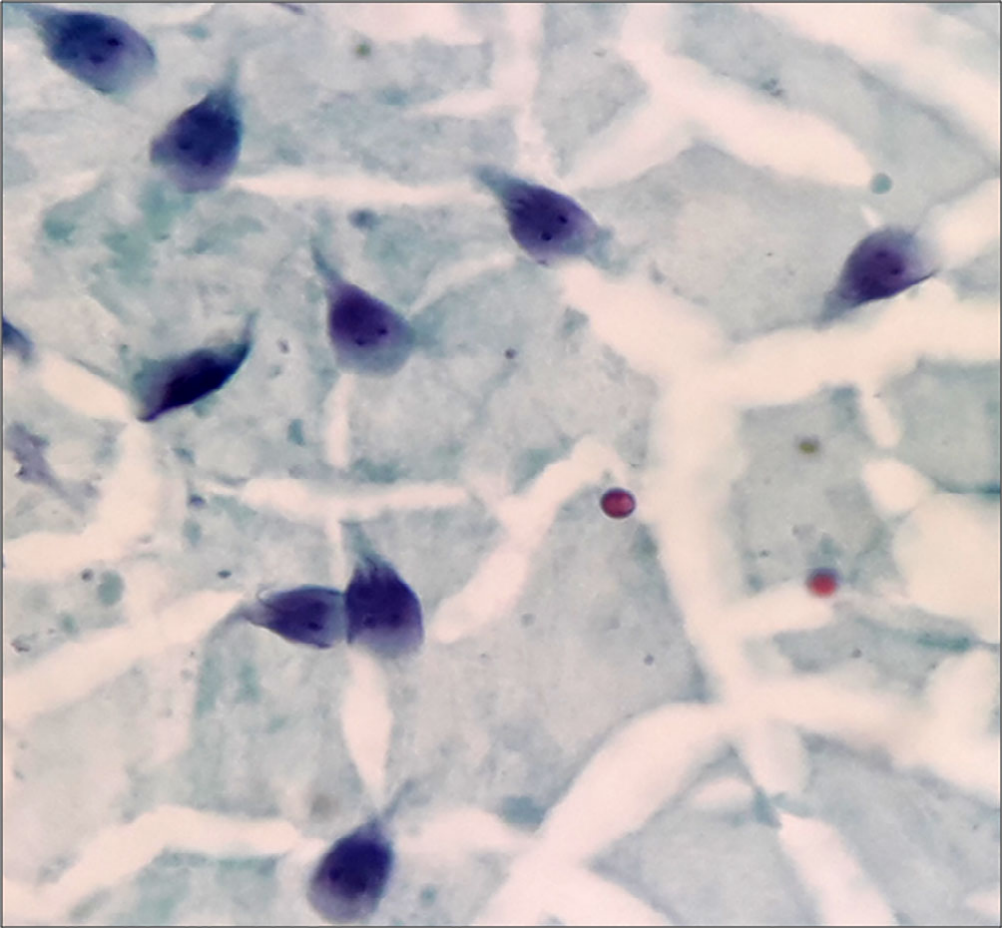
Common bile duct brushings showing pear shaped trophozoites of *Giardia duodenalis* with symmetrically placed nuclei and flagella. (Papaniculaou, ×1000)

Giardiasis is one of the commonest intestinal infections affecting individuals globally. The worldwide prevalence ranges from 1% to 65% while countries like India report prevalence as high as 46.5% to 97.4%.^1^ It is caused by *G. duodenalis* (also known as *Giardia intestinalis* and *Giardia lamblia*).

*Giardia duodenalis* has two morphologic forms: cysts and trophozoites. Cysts are excreted in stool and are infectious forms while excystation produces trophozoites in proximal small bowel. It is transmitted through faeco‐oral route. Cysts are resistant to chlorine treatment and may survive in cold water for several months.

Patient may present with self‐limiting infestation, nonspecific symptoms like diarrhea and abdominal cramps or chronic malabsorption and stunted growth. Diagnosis can be made by identification of organisms by stool microscopy or endoscopic biopsy. Other tests include nucleic acid amplification or antigen detection test. Rarely *G. duodenalis* may be detected in CBD brushings^2^ by possible contamination from duodenal contents during endoscopic retrograde cholangiopancreatography. Treatment includes antimicrobial therapy and supportive care.

## CONFLICT OF INTERESTS

None.

## AUTHOR CONTRIBUTIONS

Conceptualization: Abha Thakur

Data Curation: Abha Thakur, Haimanti Sarin

Writing ‐ original draft preparation: Abha Thakur

Writing ‐ review & editing: Haimanti Sarin

Both authors have read, edited and reviewed the manuscript.
